# Evaluation of the Hydration Characteristics and Anti-Washout Resistance of Non-Dispersible Underwater Concrete with Nano-SiO_2_ and MgO

**DOI:** 10.3390/ma14061328

**Published:** 2021-03-10

**Authors:** In Kyu Jeon, Byeong Hun Woo, Dong Ho Yoo, Jae Suk Ryou, Hong Gi Kim

**Affiliations:** Civil and Environmental Engineering Department, Hanyang University, Jaesung Civil Engineering Building, 222 Wangsimni-ro, Seongdong Gu, Seoul 04763, Korea; supermacy94@daum.net (I.K.J.); dimon123@hanyang.ac.kr (B.H.W.); dongho3461@naver.com (D.H.Y.); jsryou@hanyang.ac.kr (J.S.R.)

**Keywords:** underwater concrete, anti-washout, hydration, nano-SiO_2_, MgO

## Abstract

In this paper, the effect of nano-SiO_2_ (NS) and MgO on the hydration characteristics and anti-washout resistance of non-dispersible underwater concrete (UWC) was evaluated. A slump flow test, a viscosity test, and setting time measurement were conducted to identify the impacts of NS and MgO on the rheological properties of UWC. The pH and turbidity were measured to investigate the anti-washout performance of UWC mixes. To analyze the hydration characteristics and mechanical properties, hydration heat analysis, a compressive strength test, and thermogravimetric analyses were conducted. The experimental results showed that the fine particles of NS and MgO reduced slump flow, increased viscosity, and enhanced the anti-washout resistance of UWC. In addition, both NS and MgO shortened the initial and final setting times, and the replacement of MgO specimens slightly prolonged the setting time. NS accelerated the peak time and increased the peak temperature, and MgO delayed the hydration process and reduced the temperature due to the formation of brucite. The compressive results showed that NS improved the compressive strength of the UWC, and MgO slightly decreased the strength. The addition of NS also resulted in the formation of extra C–S–H, and the replacement of MgO caused the generation of a hydrotalcite phase.

## 1. Introduction

As bridges continue to age, experts have been increasingly concerned about their structural safety, and suggest strengthening procedures. However, structures located underwater are more difficult to strengthen than superstructures. The existing strengthening methods [1,2,3,4,5,6] for substructures are costly, traffic-disrupting, and have a long construction time. To deal with these weak points of conventional strengthening methods, the FRP underwater strengthening method [7], the jacket strengthening method [8], and the precast concrete segment assembly method [9] have been proposed. Underwater concrete (UWC) is commonly used in these methods, and its performance is important in determining strengthening efficiency [10,11].

UWC is generally produced using viscosity-modified admixtures (VMAs) and anti-washout admixtures (AWAs) to fabricate viscous concrete mixes [12]. It is directly poured into water, and washout resistance is a significant factor that determines its strength, durability, and workability. In underground engineering, UWC with anti-washout characteristics is used due to its excellent viscosity, low dispersibility, and low water pollution potential [13]. As UWC is based on self-compacting concrete, its rheological properties must be adequately maintained to ensure higher washout resistance.

Several attempts have been made to control the rheological properties and washout resistance of UWC. Park et al. [14] studied the various contents of AWA and its superplasticizer, and found that the optimum content of UWC is 1% AWA and 5.5% superplasticizer. Kumar et al. [15] mentioned that a combination of AWAs can postpone the setting time and strengthen the rheological properties of UWC. Khayat et al. [16] achieved higher anti-washout resistance using fly ash and silica fume as viscosity-enhancing agents. In addition, Grzeszczyk et al. [17] used a nanomaterial to enhance the anti-washout resistance of UWC. Sonebi et al. [18] used 8% silica fume and 20% cement binder instead of fly ash to control the workability and stability of UWC, which also decreased the segregation coefficient, washout loss, and surface settlement. Kojouri et al. [19] used lime powder as a mineral additive in UWC and found that it enhanced washout resistance, workability, the acceleration of the hydration reaction, and compressive strength.

Nano-SiO_2_ (NS) is the common name for nano-sized silica particles with a diameter between 5 nm and 100 nm. NS particles are about 1000 times smaller than OPC particles [20]. Previous studies [21,22] found that NS can strengthen the cement matrix due to its smaller particles, high pozzolanic activity, and ability to form dense and compact microstructures. Because of these properties of NS, many studies focus on enhancing the mechanical performance and durability of cement composites with different percentages of NS [23,24,25,26,27,28]. However, only a few studies [17] of NS have been used to control the washout resistance of UWC. Therefore, further specific investigations and analyses of the fluidity and anti-washout resistance of UWC with NS are needed.

Magnesium oxide (MgO) is widely used in concrete manufacturing. Previous studies have demonstrated that the hydration and expansion characteristics of MgO positively affected concrete by reducing the hydration heat, decreasing the shrinkage, and changing the porosity and pore size distribution [29,30,31,32]. As the anti-washout resistance of concrete depends on the fine fraction of its binder material, various very fine mineral additives have been suggested to control the anti-washout properties of UWC [17]. Fly ash, silica fume, and ground granulated blast furnace slag have been generally used as mineral additives in UWC mixes [33,34]. Studies have shown that the incorporation of mineral admixtures in UWC is sought around the world to produce high-quality non-dispersible UWC. Although much attention has been given to fly ash, silica fume, and granulated blast furnace slag, there are few data available on the effects of NS and MgO on the anti-washout resistance of UWC.

In summary, this study investigated the effects of NS and MgO as mineral admixtures on the workability, washout properties, strength development, and hydration process of UWC. A slump flow test and a viscosity test were conducted to analyze these effects on the workability of UWC; the pH and turbidity measurement tested the anti-washout resistance of UWC; the setting time and isothermal conduction calorimetry were used to determine the hydration characteristics of UWC; and a compressive strength test measured the mechanical property of UWC.

## 2. Experimental Details

### 2.1. Materials

**Figure 1:**
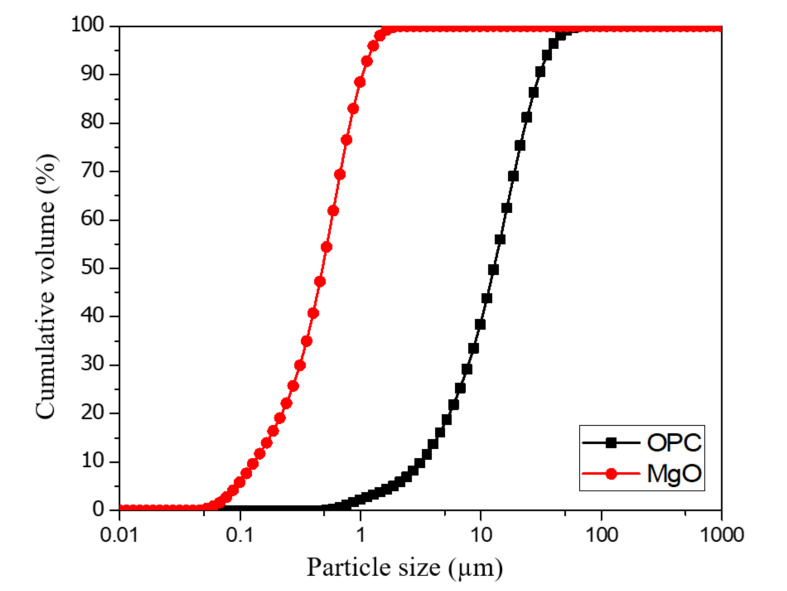
Particle size distributions of Ordinary Portland Cement (OPC) and magnesium oxide (MgO).

Ordinary Portland Cement (OPC) obtained from A company (Seoul, Korea) that complies with ASTM C-150 [35] was used as the primary binding material. Specifically, fine and coarse aggregates with maximum sizes of 5 mm and 25 mm, respectively, were used. The fine aggregate had a fineness of modulus (F.M) of 2.7 and a water absorption capacity of 1.05%, and the coarse aggregate had a specific gravity of 2.63, an F.M. of 7.01, an abrasion of 45%, and a water absorption capacity of 1.02%. To achieve the needed fluidity of UWC, a poly-carboxylate-based superplasticizer from E company in Seoul (SP) was used. Commercially available hydroxypropyl-methylcellulose (HPMC)-based anti-washout admixture (AWA) from a local E company in Seoul was used to produce non-dispersible UWC mixes. Magnesium oxide (MgO) with a purity of 80.1%, especially light burned magnesia (LBM), sourced from R company in Gwangyang, Korea was used as the binder replacement material. The gradation curves for the OPC and MgO used are shown in Figure 1. The gradation curves indicated that the MgO particles have a smaller size compared to OPC. Nano-SiO_2_ (NS) powder with a purity of 99.5% and average particle sizes of approximately 20–40 nm was used as an additional binder material.

### 2.2. Mix Proportions and Fabrication of Specimens

Five mix proportions of non-dispersible UWC and control concrete mixtures were prepared. Table 1 shows the mix proportions of all the concrete mixes produced in this study, which were classified into two types. The NS series denotes the sample with NS powder, and the M-series represents the samples with MgO. The numbers after NS and M indicate the dosage: 0%, 5%, and 10% of the weight of OPC was sequentially replaced with MgO, and NS powder was sequentially added at 0%, 1%, and 2% of the OPC weight. A water binder (Sum of OPC and MgO) ratio of 0.45, a superplasticizer content of 2% of the weight of the binder, and an AWA content of 0.5% of the weight of the binder were used, followed by a standard mix of 25 MPa target strength UWC obtained from a local concrete company. Concrete cylinders with dimensions of Φ 100 mm × 200 mm were prepared following ASTM C 31 [36]. The casting method of the anti-washout UWC was adopted from previous study [37]. The UWC specimens were demolded after they were cured for 24 h and placed in a water curing condition until the set curing age was reached.

### 2.3. Experiment Methods

The concrete mixes were prepared with a laboratory-scale concrete mixer (MR500, Inter, Korea). The fresh properties of the UWC mixes were determined by testing their workability. The slump test was conducted according to ASTM C 143 [38]. The viscosity values of the UWC mixes with NS and MgO were assessed with a rheometer (Rheometer R/S Plus, Brookfield, WI, USA). To observe the setting time of the fresh UWC mixes, paste samples were obtained after mixing the UWC concrete, and the initial and final setting times were investigated using Vicat needles [39,40] according to ASTM C 191 [41].

To measure the anti-washout resistance of the UWC mixes, the pH and turbidity measurements preceded via a method used in a previous study [42]. First, 500 g of UWC was slowly poured into a beaker. After 3 min, 100 mL of water from the top surface of the beaker was extracted using a pipette, its pH was immediately evaluated with a pH meter, and its turbidity was measured in accordance with EN ISO 7027 [43] using a turbidity machine (TB 300IR, Lovibond, Amesbury, UK).

To evaluate the mechanical properties of UWC concrete mixes, the compressive strength of the concrete cylinders with dimensions of Φ 100 mm × 200 mm at 7 and 28 days of curing was measured using a universal testing machine (UTM, Shimadzu, CCM-200A; Shimadzu Corporation, Kyoto, Japan) and following ASTM C39 [44]. Three replicates were used for each mix.

Isothermal calorimetry is a convenient means of examining the hydration characteristic of cementitious materials at early ages. The heat evolution was investigated using a semi-adiabatic calorimeter, as in a previous study [45]. The specimens were placed in the calorimeter 2 min after the water and binder were mixed. The temperature change was measured every 3 min for 48 h.

The hydration product of the UWC concrete specimens at later ages was measured via thermogravimetric analysis (TGA). The powder samples were obtained from pieces of the concrete specimens used for the compressive strength test. A thermogravimetric analyzer (TGA7 PERKIN ELMER, TA Instruments, New Castle, DE, USA) was used for TGA analysis in an N_2_ environment at a heating rate of 10 K/min within a temperature range of 2 to 1000 °C.

## 3. Results and Discussion

### 3.1. Slump Flow Test

**Figure 2:**
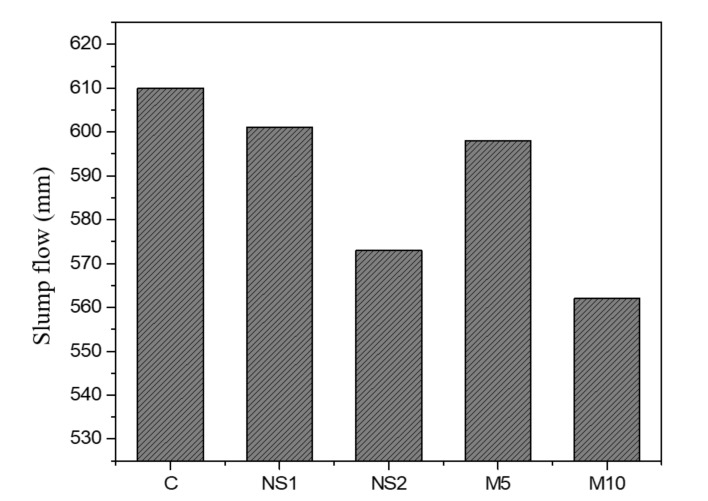
Slump flow results of the underwater concrete (UWC) mixes with different dosages of NS and MgO.

To evaluate the effects of NS and MgO on the workability of UWC concrete mixes, a slump flow test was conducted, as shown in Figure 2. The slump flow decreased with the addition of NS and its replacement with MgO. For example, slump values of 610 mm, 601 mm and 574 mm were measured for the control, NS1, and NS2 concrete mixes, respectively, mainly due to the large specific surface area of the NS powder. The fine particles of NS in the cement matrix had the strongest effect on the total water demand of the concrete mixes [46]. Therefore, the finer the particle powder added, the less workable the UWC. In addition, the incorporation of MgO in the UWC concrete mixes reduced the slump flow value. For instance, slump flow values of 610 mm, 598 mm, and 562 mm were measured for the control, M5, and M10 specimens, respectively. The decrease in fluidity with MgO was similar to the results of a previous study [47]. Finer MgO particles enhanced the cohesiveness of the cement paste, which reduced the fluidity. Furthermore, Zhang et al. [47] reported that the incorporation of reactive MgO decreased the workability of a cement composite, which increased its water demand to achieve a similar flow value.

### 3.2. Viscosity Test

**Figure 3:**
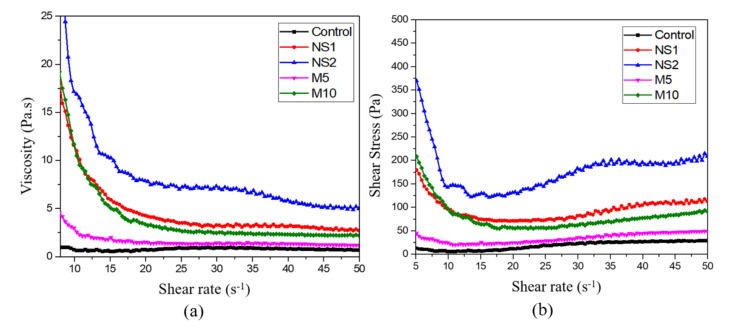
(**a**) Viscosity versus shear rate flow curves and (**b**) shear stress versus shear rate flow curves for UWC paste with different amounts of NS and MgO.

Figure 3 shows the rheological properties of the UWC mixes with different amounts of NS and MgO. Figure 3a shows the relationship between viscosity and shear rate, and Figure 3b shows the shear stress versus shear rate flow curves for the UWC mixes. Figure 3a illustrates that the enhancement of the shear rate decreased the viscosity. The addition of NS and replacement OPC with MgO increased the viscosity compared to that of the control specimens. Among the five UWC mixes, that to which 2% NS was added showed significant viscosity enhancement, followed by the NS1, M5, M10, and control specimens. Figure 3b shows that the shear stress increased when shear rate increased. In the region with a low shear rate of approximately 10 s^−1^, the shear stress was significantly reduced, probably because of destruction of the flocculated structures due to rotor rotation [48]. The shear stress results are similar to the viscosity values. As the amounts of NS and MgO increased from 1% to 2%, the shear stress improved compared to that of the control specimen. However, NS enhanced the viscosity more significantly than MgO. The higher UWC viscosity values could be attributed to the finer particle sizes with higher specific surface areas of NS and MgO, which required more water to achieve flow [49]. As NS has a much smaller particle size than MgO, its viscosity enhancement effect was greater.

### 3.3. Setting Time

**Figure 4:**
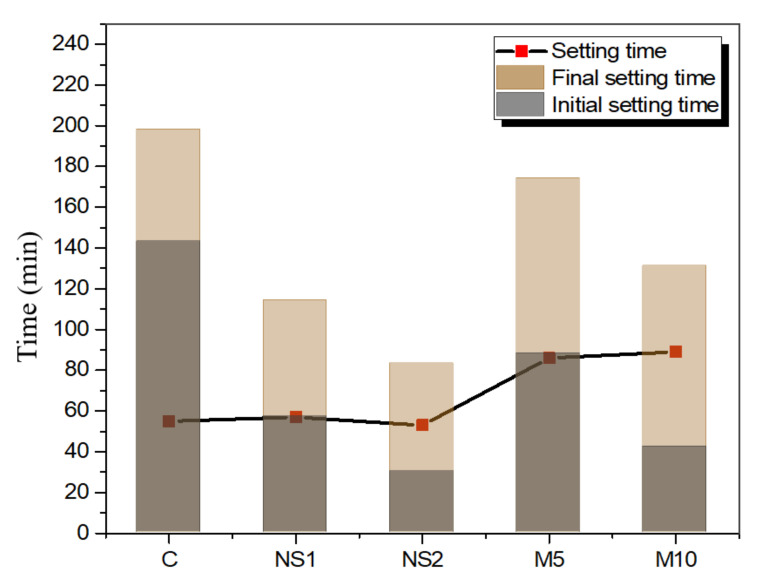
Initial and final setting times of UWC pastes with different amounts of NS and MgO.

The initial setting time, the final setting time, and the setting time that is the duration between the initial and final setting times of the UWC pastes with different NS and MgO contents, are illustrated in Figure 4. Both NS and MgO incorporation into the UWC mixes reduced the initial and final setting times compared to the control specimen. When NS was increased from 1% to 2% and when MgO was increased from 5% to 10%, the initial setting time decreased by 60.1%, 79%, 38.46%, and 70.63%, respectively, and the final setting time decreased by 42.42%, 58.1%, 12.1%, and 33.84% compared to the control specimen. The results showed that the setting time decreased as the NS and MgO contents increased. However, NS reduced the setting time more significantly than did MgO. The reduction in the setting times of the UWC mixes that contained NS and MgO might be related to the finer particle sizes and higher surface areas of NS and MgO. Zhang et al. [50] found that NS decreased the setting time by reducing the dormant period and enhancing cement hydration. Li et al. [51] reported that the addition of NS promoted the gelation of C–S–H gels, which reduced their setting time. However, for MgO, the initial setting time decreased, and the setting time improved, compared to that of the control specimen. This was due to the fine particle size and hydration properties of MgO. The finer particle size of MgO compared to that of OPC gives it a larger surface area and a higher water demand, which shortens the initial setting time. The delay in final setting time could have been due to Mg(OH)_2_ formation during MgO hydration. Polat et al. [52] found that Mg(OH)_2_ formation slowed the hydration process and delayed the setting time by encircling the cement particles.

### 3.4. Anti-Washout Resistance

**Figure 5:**
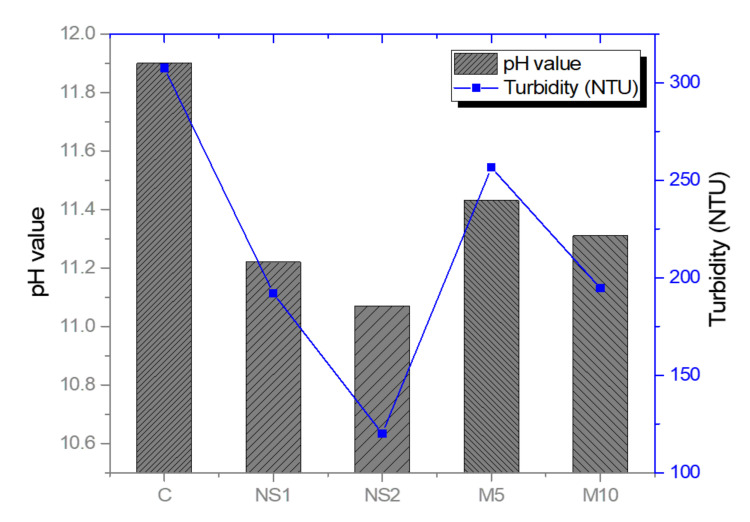
Anti-washout resistance of UWC concrete mixes with NS and MgO.

To evaluate the anti-washout resistance of the UWC concrete mixes, pH measurements and a turbidity test were conducted. Figure 5 shows the pH and turbidity values of each of the mixes. UWC generally loses quality during on-site construction because cement materials are washed off and diluted by water flow [37]. If segregation and washout occur while the concrete mixes are poured, the overall pH and turbidity of the cement particles with a high pH value increase in the water. The high pH and turbidity values of UWC concrete mixes signify their washout resistance ability. As in Figure 5, all the mixes showed similar tendencies in terms of pH and turbidity values. The results indicate that the addition of NS powder reduces the pH and turbidity values of UWC mixes. For example, pH values of 11.9, 11.22, and 11.07, and turbidity values of 307.5 NTU, 192 NTU, and 120 NTU, were reported in the control, NS1, and NS2 specimens, respectively. This can be attributed to the reduced segregation of the UWC concrete mixes by the fine particles of NS because of their cohesive effect, as mentioned above. Senff et al. [53] reported that the incorporation of NS can reduce the diameter of the spread on the flow table due to increased cohesiveness. For MgO replacement, pH values of 11.9, 11.63, and 11.31 and turbidity values of 307.5 NTU, 256.5 NTU, and 194.5 NTU were measured in the control, M5, and M10 specimens, respectively. The results showed that pH and turbidity decreased with increased MgO replacement. The increase in the anti-washout resistance of the UWC that contained MgO presumably occurred because the fine particles of MgO increased the cohesion of the concrete mixes. Both NS and MgO increased the cohesion of the UWC mixes due to their finer particle sizes compared to that of OPC, which enhanced the anti-washout resistance of the UWC.

### 3.5. Compressive Strength Test

**Figure 6:**
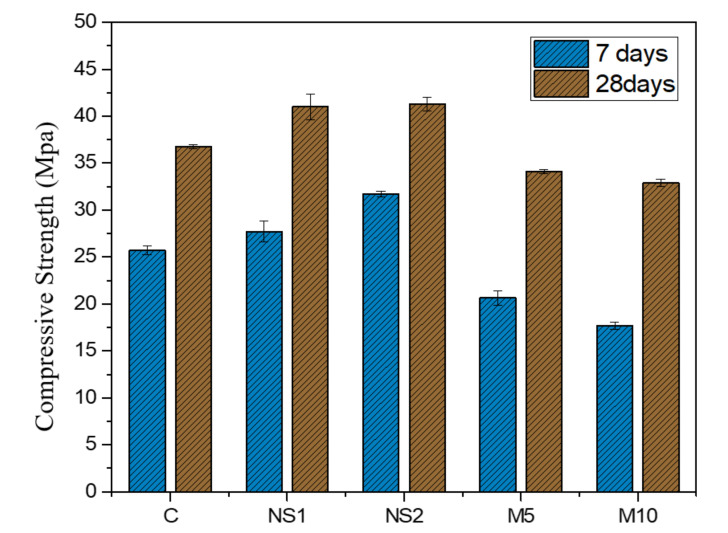
Compressive strength results of UWC concrete specimens with different amounts of NS and MgO after 7- and 28-day curing.

To investigate the effects of NS and MgO on the mechanical properties of the UWC concrete, a compressive strength test was conducted after 7 days and 28 days of curing. The results are shown in Figure 6, whereby the compressive strength increased with curing age in all mixes. This is because the continued hydration of cementitious materials increases the compressive strength. The 28-day compressive strengths attained for C, NS1, NS2, M5, and M10 were 36.73 MPa, 40.9 MPa, 41.27 MPa, 34.11 MPa, and 32.88 MPa, respectively. After 7 days and 28 days, the compressive strength of the concrete that contained NS was slightly higher than that of the concrete without NS. This strength development with NS was due to the fine particles and high pozzolanic activity of NS, which provide extra nucleation sites to cement particles, accelerate their hydration, and generate additional hydration products. Yu et al. [25] found that the application of nanoparticles can increase the physical and mechanical properties of concrete by refining its microstructure. In addition, Scrivener et al. [54] and Xu et al. [55] mentioned that nanoparticles can accelerate the hydration process, densify the microstructure, and improve the Interfacial Transition Zone (ITZ) of concrete, which decreases the porosity and enhances the compressive strength of a cement composite. However, the incorporation of MgO into UWC reduces the compressive strength of UWC after 7 days and 28 days, compared to that of the control specimen. As the MgO proportion increased from 0% to 10%, the compressive strength of M5 and M10 decreased by 7.13% and 10.48%, respectively, after 28 days of curing, compared to the control specimen. This is because the amorphous active silica in MgO can react with MgO or Mg(OH)_2_ and water during the hydration process [56,57]. Unluer et al. [58] reported that MgO reduced the compressive strength of cement due to the formation of brucite, which is weaker than C–S–H, in normal cement hydration.

### 3.6. Hydration Heat

**Figure 7:**
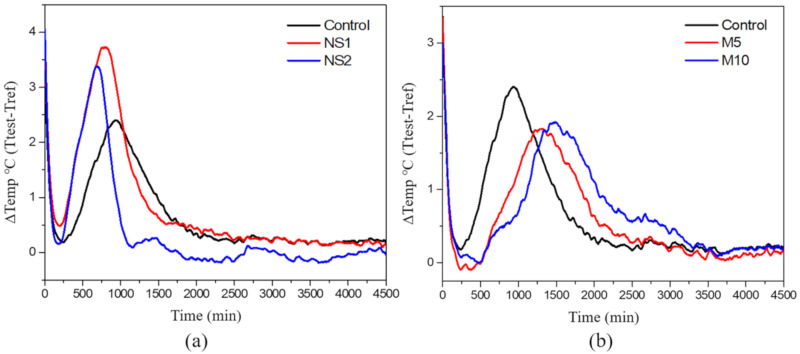
Calorimetric curves of the rate of heat release of (**a**) UWC with different NS contents and (**b**) UWC with different amounts of MgO.

The isothermal calorimetry method is used to investigate the effects of temperature on the reaction kinetics of cementitious materials [40]. Figure 7 shows the calorimetric curves of heat release from the UWC specimens with (a) NS and (b) MgO. Detailed information on peak time and peak temperature variation is listed in Table 2.

The first sharp peak was produced from the dissolution of the dry mixtures, and occurred immediately after the mixtures were mixed [59]. The second peak was generated between 500 and 2000 min and was related to the polymerization degree of the binder materials [60]. Figure 7a shows that the incorporation of NS into the UWC specimens accelerated the peak time and increased the peak temperature variation. Peak times of 940 min, 784 min, and 696 min were reported for the control, NS1, and NS2 specimens, respectively. In addition, the control, NS1, and NS2 specimens showed temperature variations of 2.4 °C, 3.73 °C, and 3.38 °C, respectively. This effect was mainly due to the seeding effect of nano-materials. Moreover, the acceleration effect of NS particles on cement hydration was due to the additional nucleation of C–S–H caused by the increased surface area of the nano-particles [61,62]. However, NS1 showed higher temperature variation compared to NS2. This phenomenon might be due to the dispersion of NS particles in the cement composite. The usage of more than adequate amounts of NS is considered to reduce efficiency due to the dispersion problem [63]. Figure 7b shows the effect of MgO content on the heat release of UWC specimens. When the amount of MgO increased, the peak time tended to be delayed, and the peak temperature was lower than that of the specimens without MgO. Although MgO has a smaller particle size than OPC, the UWC specimens that contained MgO showed lower hydration rates and heat release due to the lower reactivity of MgO. Mehta et al. [64] reported that the MgO within cement slightly delayed hydration by producing magnesium hydroxide, which is insoluble.

### 3.7. Thermogravimetric Analysis

**Figure 8:**
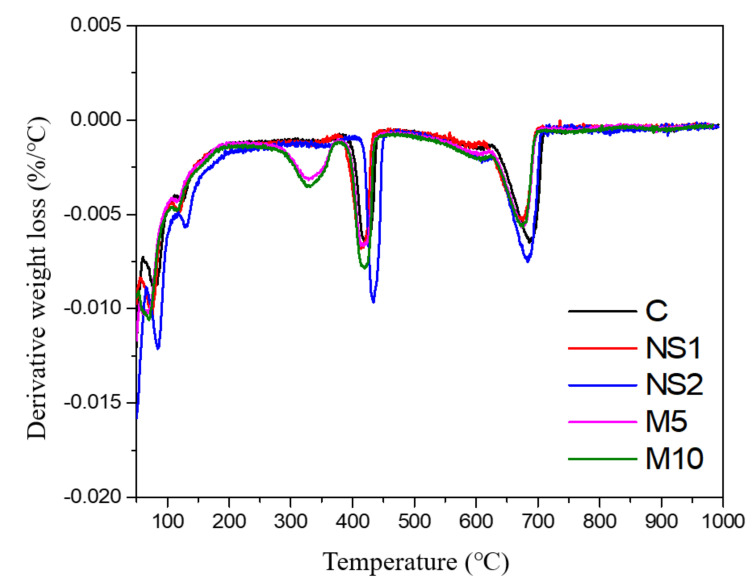
DTG curves of the UWC mixes with different amounts of NS and MgO.

Figure 8 shows the Derivative Thermo Gravimetry (DTG) curves of the UWC mixes with different amounts of NS and MgO after 28 days of curing. All the UWC mixes showed mass loss peaks in four temperature regions. The mass loss below 15 °C is attributed to the evaporation of free water from the pore structure [65,66]. Lothenbach et al. [67] found that mass losses between 50 and 200 °C were caused by the dehydration of C–S–H, and the secondary peaks at around 146 °C were associated with the decomposition of ettringite. Bernal et al. [68] and Rozov et al. [69] reported that the mass losses between 250 and 400 °C were related to the thermal decomposition of hydrotalcite. In the present study, no carbonate source was used, so the mass loss in this region was mainly due to the dehydroxylation of hydrotalcite [70]. In addition, the C–H groups at around 420 °C and between 660 and 700 °C were associated with the decarbonation of CaCO_3_. However, this carbonate region can be generated through powder manufacturing [71]. The results showed that increasing the NS content increased the mass loss when below 200 °C, as seen on the DTG curve of the UWC mixes. For specimens with and without NS, the type of hydration products remained unchanged. Only M5 and M10, which contained MgO, showed significant mass losses between 250 and 400 °C, which indicated the presence of hydrotalcite in the UWC with MgO.

**Figure 9:**
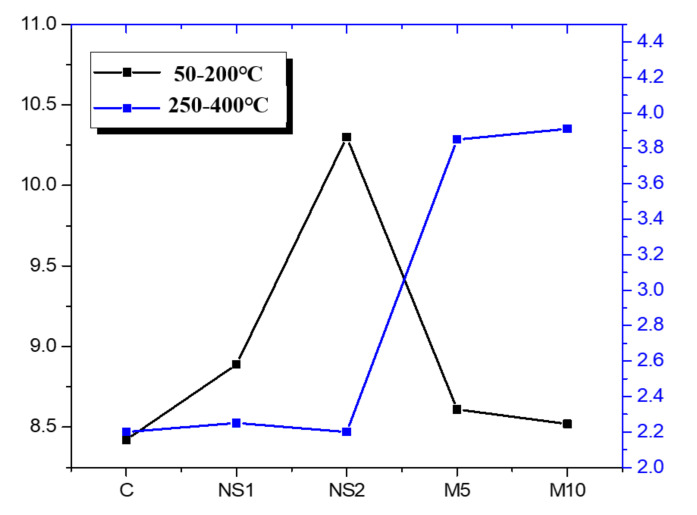
Mass loss fractions at different temperature ranges of the UWC mixes as a function of NS and MgO.

Figure 9 shows that the mass loss fractions contributed to the C–S–H (50–200 °C) and the hydrotalcite (250–400 °C) of the UWC mixtures as functions of NS and MgO. In the 50 to 200 °C region, the specimens that contained NS had higher mass loss than the control specimens. For example, mass loss fractions of 8.4%, 8.9%, and 10.3% were noted in the control, NS1, and NS2 specimens, respectively. The increase in the C–S–H gel is attributed to the promotion of cement hydration by NS due to its pozzolanic activity and nucleation effect [72]. In addition, in the 250 to 400 °C region, the specimens that contained MgO showed enhanced mass loss fractions, which led to higher amounts of hydrotalcite. A similar phenomenon was reported in a previous study. Yoon et al. [73] found that, when MgO was incorporated in a cement composite, the MgO combined with Al and promoted the generation of the hydrotalcite phase in the cement composite.

## 4. Conclusions

This study investigated the effects of NS and MgO on the hydration characteristics and anti-washout resistance of non-dispersible UWC concrete. The following conclusions were reached.

1. The results of the rheological experiments revealed that the fine particles and large specific areas of NS and MgO reduced the flowability and increased the viscosity of UWC. However, the impact of the smaller NS on the rheological properties was greater than that of MgO;
2. Both NS and MgO shortened the initial and final setting times compared to the control specimens due to their fine particle sizes. However, MgO prolonged the setting time (the duration between the initial and final setting times) due to the formation of an insoluble hydration product. The hydration heat analysis showed that NS accelerated the hydration process and increased the hydration heat, whereas MgO delayed the peak time and reduced the temperature;
3. The anti-washout performance results showed similar pH and turbidity tendencies in all the mixes. Both the addition of NS and replacement OPC with MgO reduced the pH and turbidity values, demonstrating that both NS and MgO increased the cohesion of the UWC mixes due to their finer particle size compared to that of OPC, which enhanced the anti-washout resistance of the UWC;
4. The addition of NS slightly increased the compressive strength of the UWC because the high pozzolanic activity of NS generated extra nucleation sites and accelerated the hydration process. However, MgO decreased the compressive strength of M5 and M10 by 7.13% and 10.48%, respectively, compared to that of the control specimen due to the formation of brucite;
5. The TGA results showed that NS promoted the generation of C–S–H gel due to its pozzolanic activity and nucleation effect. In addition, the specimens that contained MgO showed enhanced mass loss fractions at 25 to 400 °C, which generated larger amounts of hydrotalcite.

## Figures and Tables

**Table 1:** Concrete mix proportions.

Concrete Mix	OPC (kg/m^3^)	MgO (kg/m^3^)	Nano-SiO_2_ (kg/m^3^)	Fine Aggregate (kg/m^3^)	Coarse Aggregate (kg/m^3^)	AWA (kg/m^3^)	SP (kg/m^3^)	W (kg/m^3^)
Control	440	–	–	708	889	2.4	7.92	198
NS1			4.4					
NS2			8.8					
M5	418	22	–					
M10	396	44						

**Table 2:** Isothermal details of the UWC specimens with NS and MgO.

Concrete Mix	Peak Time (min)	Peak Temperature (∆ °C)
**Control**	940	2.40
**NS1**	784	3.73
**NS2**	696	3.38
**M5**	1222	1.81
**M10**	1492	1.92

## Data Availability

Data sharing is not applicable.
